# Unraveling Pathway Complexity in Photoreversible Supramolecular Hydrogels: Real‐Time Visualization and Sub‐Micrometric Photopatterning

**DOI:** 10.1002/smll.75208

**Published:** 2026-08-16

**Authors:** Federico Movilla, Consuelo Ripoll, Cristián Huck‐Iriart, Juan A. González‐Vera, Angel Orte, Rafael Contreras‐Montoya

**Affiliations:** ^1^ Nanoscopy‐UGR Laboratory, Departamento de Fisicoquímica Unidad de Excelencia de Química Aplicada a Biomedicina y Medioambiente Facultad De Farmacia Universidad De Granada, Campus Cartuja Granada Spain; ^2^ ALBA Synchrotron Light Source, Cerdanyola del Vallès Barcelona Spain

**Keywords:** fluorescence lifetime microscopy, pathway complexity, photopatterning, photoreversible gels, supramolecular hydrogels

## Abstract

Photoreversible supramolecular hydrogels hold immense potential as adaptive materials, yet the microscopic mechanisms governing their light‐triggered disassembly and reassembly remain poorly understood, limiting their technological application. Here, we unravel the pathway complexity of an arylazopyrazole‐based hydrogel using a novel multidimensional methodology combining Fluorescence Lifetime Imaging Microscopy (FLIM), SAXS, and Molecular Dynamics. Contrary to the assumption of full solubilization, we reveal that disassembly proceeds via the fragmentation of supramolecular clusters. Furthermore, we demonstrate that the reassembly process follows a different pathway, resulting in “scar‐like” structures rather than restoring the original network. Leveraging this pathway complexity, we achieve maskless, sub‐micrometric photopatterning within the hydrogel matrix, establishing a new protocol for writing complex functional designs into soft matter with high spatiotemporal resolution.

## Graphical abstract

This research demonstrates real‐time visualization of supramolecular sol‐gel transitions using Fluorescence Lifetime Imaging Microscopy (FLIM). By leveraging 5D visualization, integrating 3D space, time, and polarity resolution, we achieved maskless sub‐micrometric photopatterning within the gel matrix. Findings reveal that disassembly and reassembly occur via cluster fragmentation, with a clear pathway complexity that arises from distinct starting points ruled by specific triggers.

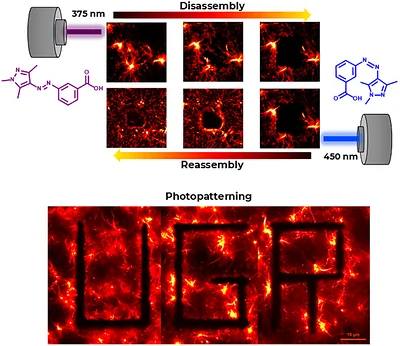

## 1 Introduction

Hydrogels are well‐known materials familiar to the general public because they are ubiquitous in modern life, present in routine products from the ink in our pens to the jelly on our plates. They can be defined as composite materials resulting from the combination of a solid and a very much larger proportion of water. The solid part, known as the gelator, generates a continuous three‐dimensional network that retains the fluid due to surface tension, making them mechanically solid‐like, even though they are composed almost entirely of water [1]. Supramolecular hydrogels are a fascinating class of soft materials formed by low‐molecular‐weight gelators (LMWGs) or supramolecular polymeric gelators that self‐assemble into a three‐dimensional network through noncovalent interactions, typically hydrogen bonding, π–π stacking, metal coordination, hydrophobic effects, and van der Waals forces [2, 3]. Unlike covalent polymers, which once formed define fixed architectures and inalterable properties, supramolecular polymers reside in a dynamic equilibrium where monomers are continuously exchanged. This reversibility translates into a unique adaptability and responsiveness, characteristics that have rendered these materials particularly attractive for applications such as self‐healing coatings, stimuli‐responsive actuators, smart biomedical devices, among others [4].

Photoreversible supramolecular hydrogels are a special class of stimuli‐responsive materials that include light‐sensitive functionalities into the gelator that undergo reversible structural changes upon irradiation [5]. If those changes affect the gelator's ability to self‐assembly, radiation might trigger alterations on the mechanical characteristics of the hydrogel. The most interesting case is the limit scenario in which irradiation under different wavelengths can trigger the reversible transitions *gel‐to‐gel* and *sol‐to‐gel*. Such non‐invasive external control is especially attracting for advanced soft‐materials applications, and has recently sparked their development, yielding some remarkable examples [6, 7, 8, 9, 10].

Nevertheless, truly photoreversible supramolecular hydrogels remain scarce. Although there is extensive knowledge on how to incorporate photoreversible molecular motifs (e.g., azobenzenes, spiropyrans, diethienylethenes, stilbenes) into gelators [5], the rational design of such materials requires a deeper understanding of the mechanisms governing light‐induced supramolecular polymerization. One of the main challenges when rationally designing a supramolecular building block for these new materials is to consider that the development of a spanning network that can immobilize solvents, and the self‐assembly between building blocks to form a crystalline structure are both processes governed by the presence of noncovalent interactions [11, 12]. Thus, there is a delicate balance for a low‐weight molecule to behave as a gelator or as a crystal building block. A balance that could be tuned by changing the experimental conditions during system decomposition and phase formation [13], setting the ground for a particularly intricated pathway complexity [14].

At present, elucidating these mechanisms typically involves the integration of multiple analytical techniques that provide indirect insight into the underlying processes, with efforts focused on the development of methodologies capable of directly monitoring them. These include techniques particularly sensitive to such phenomena, such as circular dichroism spectroscopy when the gelator is chiral [14, 15], or fluorescence lifetime microscopy (FLIM) employing solvatochromic probes responsive to supramolecular aggregation [16, 17]. Recently, Kimura et al. reported the use of atomic force microscopy (AFM) to propose a novel mechanism of supramolecular gelation based on the cooperative binding of fragments to grow supramolecular polymers, as opposed to the traditional accepted models involving the assembly of individual molecules [18]. Despite these advances, a complete, direct visualization of every mechanistic step in real time remains elusive [3, 19]. Thus, the quest for a universal, time‐resolved high‐resolution method to directly observe these processes at the molecular level continues.

Among the interesting features that photoreversible soft materials present, their photopatterning capability has attracted significant attention. Recent progress in this field has enabled researchers to modify soft and supramolecular materials with unprecedented precision using light as a noninvasive stimulus [20]. With applications ranging from tissue engineering scaffolds to dynamic optical elements and flexible electronics, these techniques offer new strategies for creating adaptive, bio‐instructive, and reconfigurable materials [21, 22]. Nevertheless, implementing photopatterning in supramolecular materials at a microscopic scale faces several practical challenges. High‐resolution methods like multiphoton lithography are often slow and expensive, making them difficult to scale up. In contrast, mask‐based approaches are limited by light scattering and diffraction, which constrain the achievable feature size and depth [22, 23]. Therefore, being able to develop novel strategies to extrinsically control the self‐assembly process of small molecules is a fascinating feature not only to further extend our understanding (control) of these phenomena, but also to rationally design new technologies for miniaturization, such as sub‐micrometric phototriggered patterning on soft matter to carve complex designs inside bio‐compatible matrixes [24].

In this work, we present an interdisciplinary and robust methodology for the understanding of the mechanism involved in the supramolecular polymerization processes on photoreversible supramolecular hydrogels based on an arylazopyrazole‐based supramolecular hydrogel, 3‐((1,3,5‐trimethyl‐1H‐pyrazol‐4‐yl)diazenyl)benzoic acid (**AAP**) [25]. This system is capable of developing both *sol‐to‐gel* and *gel‐to‐sol* transitions when irradiated with light of different wavelengths in a timescale of minutes, what made hydrogel an ideal candidate for a time‐resolved study of the disassembly and reassembly processes.

In order to do that, we developed a novel interdisciplinary approach based on FLIM microscopy to track polarity changes during the photoinduced disassembly and reassembly process, employing a rationally designed probe ([9‐(azetidin‐1‐yl)‐5‐butyl‐4Hbenzo[*de*] [2,6]naphthyridine‐4,6(5*H*)‐dione: **AQui**), complemented with molecular dynamics simulations and dynamics small angle x‐ray scattering (SAXS) experiments. This cutting‐edge microscopy methodology granted us the capability to simultaneously visualize while phototriggering the processes synchronically, allowing us to capture the process in real time with nanometer spatial resolution and polarity‐based contrast [26]. Lastly, we present a novel technique to photopattern this material based on the use of confocal microscopy achieving sub‐micrometric spatial resolution without the requirement of masking or other accessories.

## 2 Results and Discussion

### 2.1 Photoreversibility Controlled and Observed by Confocal Microscopy

The system studied in this work is a reported hydrogel [25] capable of developing both *sol‐to‐gel* and *gel‐to‐sol* transitions when irradiating with light of different wavelengths due to changes in the supramolecular network developed by the gelator (**AAP**) as a consequence of the interconversion between two conformers (*Z* and *E*) that can be selected by direct photoexcitation at 365 nm for *E*‐*to‐Z* transitions and 520 nm for the reciprocal conversion (Figure 1). Disassembly and reassembly experiments were carried out in preformed **AAP** gels by employing a low‐intensity, mild irradiation on a square surface, focusing a 375 nm, for disassembly, or 450 nm, for reassembly, laser beam through the microscope's objective and raster scanning a 15·15 µm^2^ area. The gel samples were prepared by two different acidification methods: slow acidification using glucono‐δ‐lactone (GdL) hydrolysis and rapid acidification by adding HCl [25]. Although both methods gave place to appreciable different sample aspect (Figure 1 for GdL and Figure S3 for HCl samples), powder x‐ray diffractions patterns of both showed them to be crystallographycally identical (Figure S1). Even more, when comparing these two patterns against the simulated pDRX pattern for the single crystal of the neutral form of **AAP**, their are also in great agreement, what is a great indicator of a conserved polymorph over the three samples. It is important to highlight that an exhaustive characterization of these hydrogels, including rheology measurements, spectroscopical characterization, and different performance test over multiple phototriggered cycles could be found in the original work of Larik et al. [25]. and will not be discussed herein, as their not part of the objectives of this work.

**FIGURE 1:**
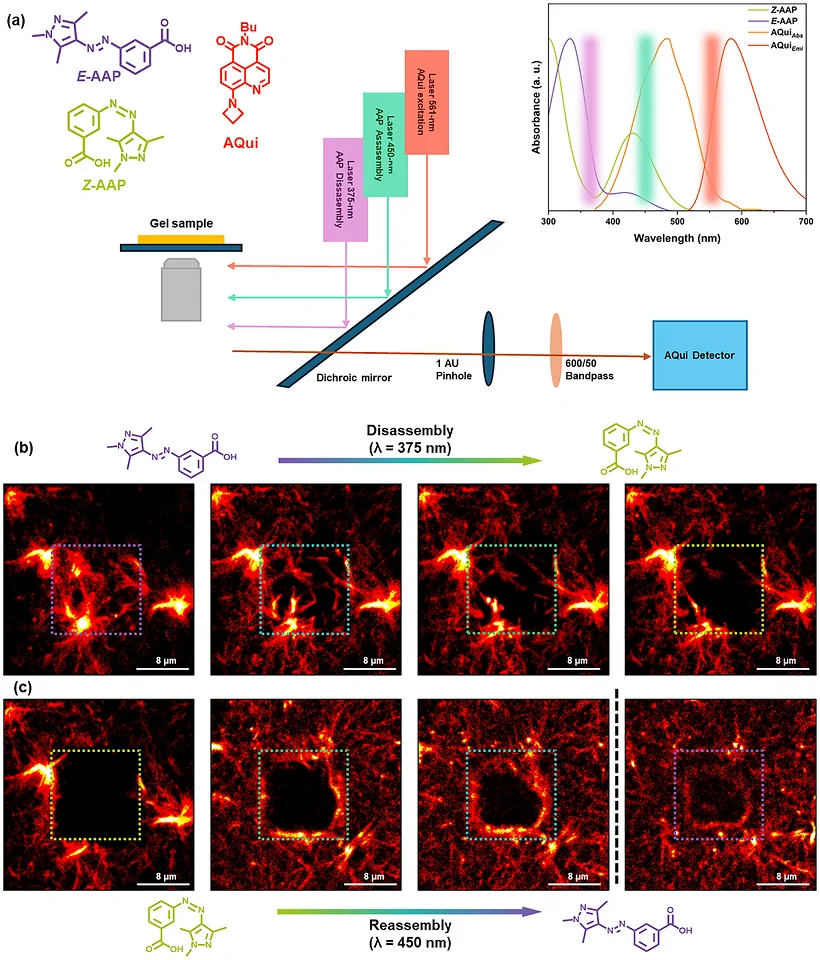
(a) Scheme of the instrumentation employed to orthogonally induce the phototriggered isomerization while capturing the emission from the probe. The absorption spectra of an aqueous solution of *Z*‐**AAP** (green), *E*‐**AAP** (purple), and the absorption (light orange) and emission (dark orange) spectrum of the probe **AQui** in DMSO are depicted in the inset, with the corresponding chemical structure of the probe and the gelators’ both isomers. (b) and (c), Phototriggered disassembly and reassembly of a GdL‐prepared **AAP** hydrogel dyed with **AQui** (50 µM, λ*_ex_* 561 nm). (b) Disassembly irradiation time‐lapse [c.a. 5’]. (c) Reassembly irradiation time‐lapse [total irradiation time 45’. The last frame was irradiated a total of 15’ without interruption to check for scar disappearance. For both rows, the section irradiated with 375 nm or 450 nm is highlighted with a colored square.

To elucidate the mechanism of such phase transitions, we employed an environment polarity sensing probe, **AQui**, specially designed for FLIM microscopy ($\lambda_{Abs}^{Max}$= 485 nm, $\lambda_{Emi}^{Max}$= 584 nm in DMSO at room temperature) [16, 26]. The lack of spectral overlap between **AQui** fluorescence and the wavelengths required to trigger both photoisomerizations ensures full photochemical orthogonality, allowing each process to be activated without perturbing the others (Figure 1a). The reporter fluorophore, **AQui**, allowed collecting both intensity and FLIM images upon exciting with 561 nm immediately after each irradiation (for a detailed description of the imaging protocol, see the Experimental Section 4.3). This soft‐irradiation method led to a controlled rupture of the gel fibers and allowed us to visualize the morphological changes and the degradation of the fibrillar network in detail during disassembly.

To control the potential intervention of the probe in the self‐assembly process, we performed ^1^H NMR experiments during the slow acidification with GdL of two different samples, one with the same amount of probe used for the microscopies, and one without it (Figure S2). This experiment showed that not only do multiplicity and coupling constants of NMR signals seem to be unaltered by the presence of the probe but, also, the time required for the signal disappearance, a value directly related to the gelification kinetics, is almost the same for both cases (c.a. 55 min).

Figure 1b shows the disassembly process of a gel of **AAP** formed employing GdL, triggered by irradiation with 375 nm light. The probe **AQui** was added to the gel to provide the necessary contrast to image the gel fibers. Thanks to the precise spatial resolution and small focus of the confocal microscope, we were able to irradiate small regions within the hydrogel. This generated hole‐like structures with well‐defined edges in a tidy delimited region of space, strongly resembling the precision of a laser‐carved pattern on other materials. To our knowledge, while there have been reported cases of self‐assembly observed with this technique [16], this is the first documented instance of supramolecular disassembly observed in real time with this level of tridimensional spatial resolution (Videos S1–S4).

Changing the irradiation wavelength to 450 nm triggered the reassembly process, which resulted in the filling of the generated void. However, there was no appreciable regeneration of the previously formed, mature fibers (Figure 1c). On the contrary, the reassembly process generated an accumulation of mass over the edges of the void, resembling the formation of a scar. This “wound‐healing” aspect is reinforced by the net mass migration observed from the gel matrix into the gap, which strongly resembles the *durotaxis* observed during biological tissue regeneration. Similar results were obtained with hydrogels formed using HCl acidification (Figure S3), although the rapidly formed hydrogel showed less defined fibrillar structures. However, although these initial results provide merely morphological information, the fluorescent probe **AQui** could also report on the polarity of the environment through its fluorescence lifetime, τ_f_, as will be presented later. This new layer of information provides important insights into the mechanism of the disassembly‐reassembly process.

### 2.2 Photopatterning Inside the Gel Matrix

One of the great advantages of confocal microscopy over other illumination techniques is its ability to focus on a small volume with minimal disturbance to the rest of the sample. By combining the spatial resolution of confocal microscopy with a rationally selected photoreversible supramolecular gelator, we developed a technique for photopatterning the gel matrix with a spatial resolution of 1 µm (Figure 2). In contrast to other described supramolecular gel photopatterning strategies [27], our technique allows sub‐micrometrical photopatterning within a supramolecular matrix that does not require the assistance of a mask. The fact that this technique requires no external attachment for photopatterning makes it more akin to well‐known laser engraving than other methods, enabling direct maskless photolithography in soft matter while overcoming the diffraction limits of mask‐based approaches. This opens the door for creating more intricate patterns that can be corrected or selectively altered by using multiple wavelengths simultaneously. This new maskless photopatterning methodology expands the possibilities for developing novel microfluidic environments on biocompatible hydrogels that can be opened to direct the diffusive flow of substrates.

**FIGURE 2:**
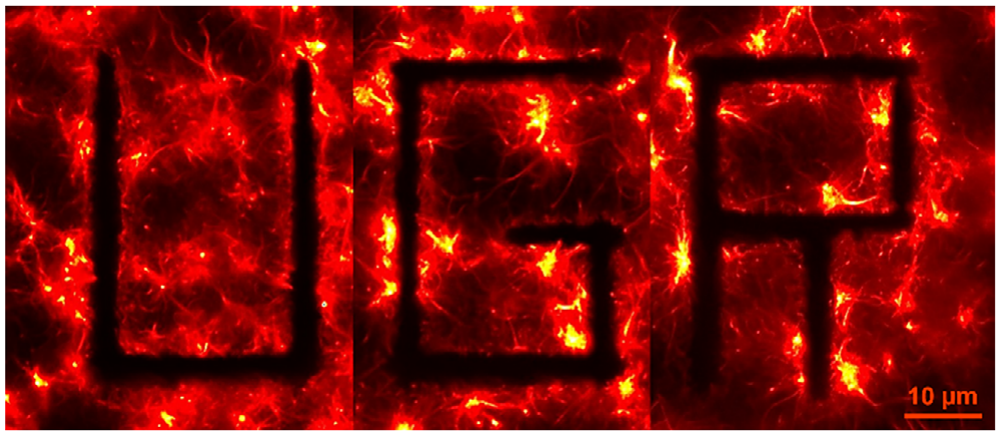
Photopatterning of the University of Granada acronym (UGR) inside the gel matrix employing a 375 nm laser‐light source**. AAP** hydrogel sample was prepared by GdL acidification and visualized by dying with **AQui** (50 µM, λ_ex_ 561 nm).

To characterize the quality of this photopatterning method, we performed a qualitative analysis of the width on different sections of the pattern, and a characterization of the edge's textures by representing the changes on the intensity along the surface cross‐section of a carved area (Figure S4). Thanks to the dynamic nature of confocal microscopy that enables real‐time observation of photopatterning, it was possible to directly observe that partially broken fibers oriented towards the interior of the carving generate a rugose, brush‐like texture over the edges. Moreover, although the carving process itself is confined to a small volume due to the confocal technique employed, small changes in local tension generated by the rupture of the solid matrix induce the re‐orientation of the fibrils, artificially generating texture and altering the final incision width after the process. This is clearly reflected in the stochastic nature of the changes in intensity that are observed when moving along a carved area (Figure S4). Interestingly, a detailed analysis of these changes shows that intensity is not lost in an abrupt manner but rather decreases gradually along the perpendicular cross‐section until no signal is observed. This behavior is consistent with the idea of a spherical focal volume with soft edges where the light intensity gradually decreases as distance from the center increases.

Besides the noisy and intricated nature of these edges, employing an adjacent‐averaging smoothing of the intensity along the cross‐section allows us to obtain an average width of 3.6 ± 0.2 µm (Figure S5).

### 2.3 FLIM Imaging During Disassembly and Reassembly

Introducing the lifetime dimension through the use of FLIM microscopy, we studied the polarity changes of the microenvironment, since the average τ_f_ of **AQui** increases with the surrounding hydrophobicity. Although this technique has been used in the past to report on different supramolecular‐ruled phenomena like amyloidogenic peptide‐peptide interactions and dipeptide hydrogel formation [16, 17, 28], to the best of our knowledge, there are no precedents of FLIM imaging employed to measure changes in polarity of a gel matrix during phototriggered disassembly and reassembly processes. To ensure reliable single‐pixel τ_f_ estimations for FLIM analysis, the gel samples were prepared using HCl rather than GdL, since the rapid gelation process avoids the formation of solid agglomerates that could introduce spurious light scattering and bias the lifetime results. Although a set of supramolecular characterizations has been previously presented to account for the role of the probe during the self‐assembly, to guarantee that the visualization wavelength combined with the presence of **AQui** does not disrupt, trigger or alter in any way the photoresponsiveness of the system, we performed two control experiments. First, a freshly prepared sample containing 10 µM of **AQui** was irradiated over 10’ uninterruptedly with 561 nm. Second, another independently prepared sample with the same proportion of AQui but photopatterned over a small area was also irradiated under the exact same conditions. In neither case did the system showed any appreciable changes in morphology or decay time (Figure S6).

During disassembly, when a fresh sample is irradiated with 375 nm light, the rupture in the material comes with a decrease in the polarity indicated by the increased lifetime of the probe (Figure 3a). Nonetheless, when reassembly is triggered with 450 nm light, the process does not appear to involve an ordered and well‐defined gel‐fiber growing that restores the previously formed structure. Instead, it is characterized by a more isotropic filling of the empty space with no appreciable changes in sample polarity (Figure 3b). As discussed above (Section 2.1), this behavior, which is consistent with what is observed for the GdL‐generated samples (Figure 1b), could arise by a combination of mass accumulation on the void's walls and a net mass migration from the gel to the void. This flux likely results from a strain gradient caused by local mechanical stress changes from this accumulation. It is important to point out that this reassembly process does not seem to imply any changes in the probe's decay time during the “healing” phase, as could be clearly seen when analyzing the distribution of decay times over the irradiated zone (Figure 3c). This result strongly contrasts to what is observed during disassembly. Although the system seems to be fully regenerated, after a first increase in the decay times only a slight subsequent decrease was observed during reassembly, accompanied by a moderate broadening of the Gaussian distribution. Interestingly, despite the isomerization process being able to assume only two possible states, the regenerated gel structure for none of the materials studied appeared identical to the fresh sample. This is particularly noteworthy for the GdL‐generated gel due to its fibrous nature.

**FIGURE 3:**
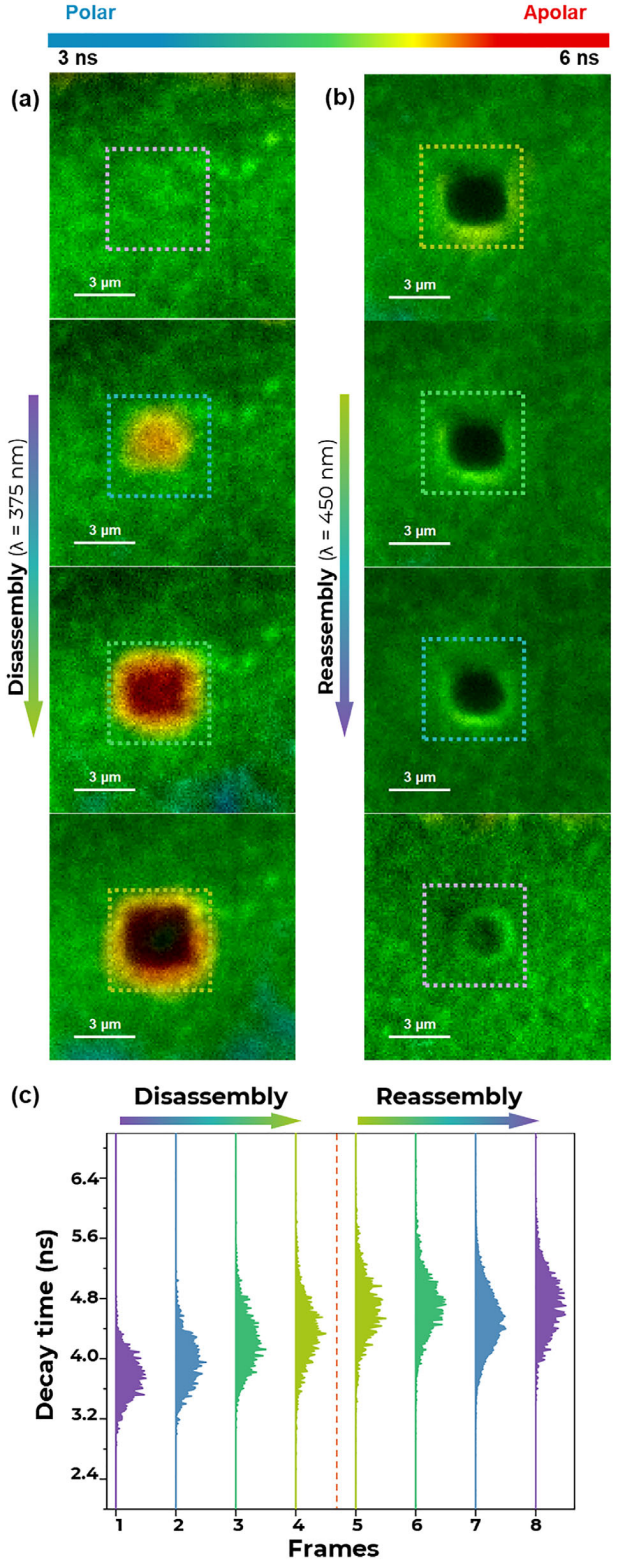
FLIM imaging of **AQui** (50 µM, λ_ex_ = 561 nm) in an HCl‐prepared **AAP** gel, during (a) phototriggered disassembly and (b) reassembly. Every frame was taken after 3’of irradiation with the corresponding 375 nm or 450 nm light source. The pseudocolor scale indicates the average τ_f_ of **AQui**, and its relationship with the environment polarity is indicated for clarity. (c) Decay time distributions computed from the irradiated square (highlighted in dashed colored lines) over the whole cycle of disassembly and reassembly.

As it is clearly visible in the images, phototriggered reassembly involves the accumulation of mass over the edges and walls of the void, resembling the formation of a scar, but not fully regenerating the fibrils. This “scar” could not be fully erased even after long irradiation times (c.a. 15’), a finding that held true even for the more isotropic and less crystalline HCl‐generated material. It is interesting to notice that the system's inability to fully recover to its original state after phototriggered reassembly has also been observed previously in an indirect way by Larik et al. [25]. They detected changes in selected ^1^H‐NMR signals during photoswitching and suggested that these changes could be due to the system being formed through different kinetic pathways when comparing photoswitched and pH‐switched systems [25]. Thus, we believe that our work not only supports this hypothesis but also provides a fundamental insight into the pathway complexity involved in the self‐assembly process of this system. It also highlights the fundamental role that crystallization‐like phenomena or other competitive mechanisms that may branch from the pathway complexity have in the availability of molecules for self‐assembly [29, 30]. This, in turn, alters the kinetics of supramolecular polymerization and directly impacts the development of the spanning network.

### 2.4 Elucidating the Self‐Assembly/Disassembly Mechanism

To gain more insight into the mechanisms of the phototriggered disassembly and reassembly of these supramolecular polymers, we complemented FLIM imaging with an interdisciplinary approach including SAXS experiments and molecular dynamics simulations. To set the ground of this study, we characterized the origins of the system before any photoirradiation. In order to do that, we conducted SAXS measurements on a freshly prepared GdL‐generated gel sample during supramolecular polymerization, revealing two distinct behaviors (Figure S7). First, a short phase (from the beginning until approximately 175 s) was marked by an average decrease in the size (*R*) of the assemblies. Second, the initial stage was followed by a long, sustained phase where the assemblies appeared to grow asymptotically. Interestingly, the fractal dimension (*D_Fr_*) during both temporal phases increased monotonically from an initial value of approximately 2.5 up to a maximum of nearly 2.9. This bimodal behavior can be attributed to a classical nucleation‐and‐growth (N&C) supramolecular polymerization process [31]. In this model, an initial phase involves the generation of a large number of small, compact, and ordered nuclei, which leads to an overall decrease in the average size due to the compacting effect that the formation of a partially ordered structure requires. The formation of tightly packed and highly ordered supramolecular structures, formed due to the strong affinity between neutral **AAP** molecules in either both conformers, is a critical step in the process, as confirmed by Molecular Dynamics (MD) simulations (see discussion below). Once this phase concludes, the generated nuclei begin to grow, increasing both the average size and the fractal dimension of the entire system.

Once the hydrogel is obtained and irradiated with 375 nm light, the microscopy images show a void that exhibits absence of **AQui** emission. This point could be considered as the ‘fully disassembled’ material (Figure 1b and Figure 3a, last panel of the first column). To better understand these changes in polarity, we performed MD simulations of mixtures with different ratios of *E*‐**AAP** and *Z*‐**AAP** in the presence of **AQui** molecules to study the nature of their interaction. The simulations showed that the probe has a clear preference for the gelating *E* isomer (Figure 4a–d). This finding suggests that the FLIM results could be biased, as the probe seems not to interact effectively with *Z*‐rich assemblies to produce a measurable signal (Figure 4d). During the whole simulation, the probe is located over the surface of the supramolecular architecture, strongly interacting with the solvent and ‘jumping’ from one cluster to another without presenting any specific interaction or affinity. Additionally, the solvatochromic effect on which the probe is based is strongly dependent on the rigidity of the surroundings [26]. Since the non‐gelating *Z* isomer forms dynamic assemblies that exhibit high interchange rates and strong interactions with the solvent, this may also inhibit the probe's sensing capability, since its emission is very low in aqueous medium [26]. Therefore, the lack of intensity in the irradiated zone may not suggest a complete disassembly process but a fragmentation of the original supramolecular assembly until the probe is in much closer contact with the external solvent and thus turns off.

**FIGURE 4:**
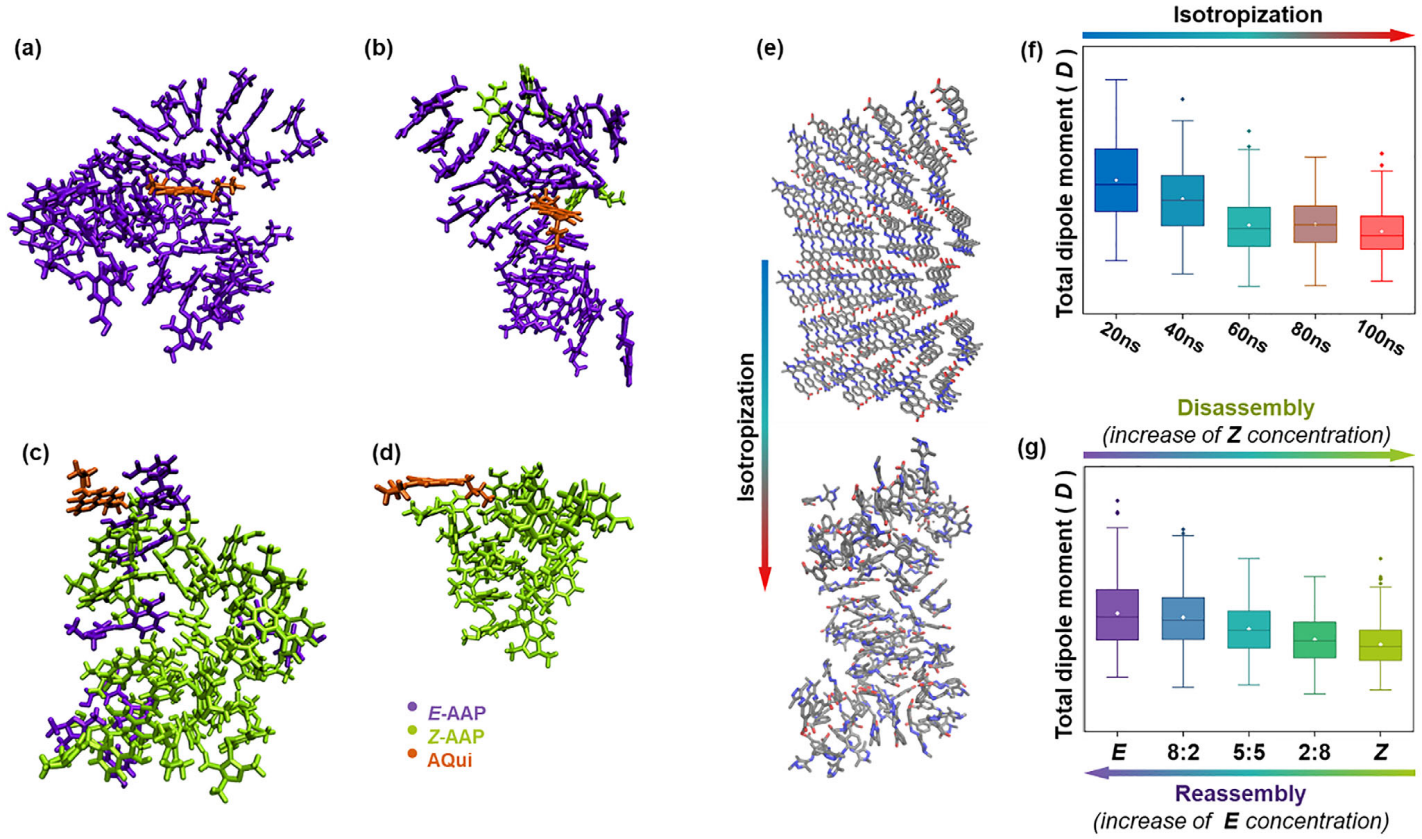
Molecular Dynamics simulations on polarity evolution of *Z*‐**AAP** and *E*‐**AAP** aggregates. (a–d), Selected supramolecular clusters generated during the molecular dynamics simulation of the self‐assembly of systems composed of different proportions of *E*‐**AAP** and *Z*‐**AAP** isomers (a: 100% *E*‐**AAP**, b: 80% *E*‐**AAP** / 20% *Z*‐**AAP**, c: 20% *E*‐**AAP** / 80% *Z*‐**AAP**, d: 100% *Z*‐**AAP**) and one molecule of **AQui**. (e) Selected structures extracted from the first and last stages of the MD simulation performed over a portion of the crystalline structure of **AAP** in water. (f and g) Boxplots (Boxes represent the interquartile range 25^th^ to 75^th^ percentiles). The horizontal full line indicates the median, while the white rhomboid represents the mean. Whiskers extend to the maximum and minimum data points within 1.5 times the interquartile range. Outliers’ data points lying beyond this range are plotted individually) computed from the total dipole moment calculated during the MD simulation of a crystalline portion in water (f) and from simulations over systems with an increased proportion of the *Z*‐**AAP** isomer (g).

The strong difference in affinity between the *E* and *Z* isomers for the probe also accounts for the polarity changes observed during the disassembly and reassembly processes. As previously discussed (Section 2.3), the disassembly process is characterized by significant polarity changes that the probe can sense. In contrast, the system's polarity during the entire reassembly process appears unchanging. This difference can be explained by the probe's starting environment in each process. At the beginning of disassembly, the probe is intercalated within the gel fibers, allowing it to sense every change in the surrounding polarity until its cavity breaks down and it is released into the aqueous medium. Conversely, upon triggering reassembly, the probe in the aqueous cavity can only effectively interact with solvent‐exposed **AAP** molecules, maintaining a dynamically changing high‐polarity environment. It is important to point out that, although the formation of a new *Z*‐**AAP** supramolecular entity in solution seems to be spontaneous, it may not be large enough to effectively intercalate a probe's molecule inside due to the large free energy barrier associated with phase nucleation. Moreover, this phenomenon is even less likely if the system presents zones that could act as growing points where the *Z* isomers could be constantly anchored and precipitate. Consequently, during re‐assembly, the probe can only report on the gelator molecules when they are fully integrated as part of the crystalline growing fibers as *E* conformers. Lastly, because this system exhibits a strong crystalline nature, the probe is not expected to sense any polarity changes while inside the crystal, which explains the homogeneous distribution of decay times over the entire irradiated zone while reassembling the system.

To study the effect of entropic forces arising from the solvent on the small fragments generated by UV irradiation during disassembly, we performed MD simulations of crystal portions in a water box (Figure 4e). From these simulations, two key features were extracted. First, the self‐assembly is not lost when the crystal is submerged; gelator molecules are not released into the solution but remain assembled throughout the whole simulation, only altering their relative position. Second, these assemblies do change their architecture, becoming more isotropic by losing some stacking interactions and relaxing the H‐bonded supramolecular network. These alterations are crucial for explaining the observed changes in polarity during the disassembly process. A more isotropic assembly implies a decrease in the overall ordering of the molecular dipole distribution, which leads to a net decrease in the total dipole moment. Dipole moment calculations over time confirm that the average total dipole moment decreases as the simulation progresses until it reaches an asymptotic value (Figure 4f). Furthermore, when MD simulations were performed on mixtures of *E* and *Z* isomers at different proportions, it became clear that even though the *Z* isomer has a theoretically higher dipole moment than *E* (7.45 D vs. 7.07 D from def2‐TZVP/B3LYP‐D4 DFT calculations), an increase in the concentration of *Z* also decreases the overall molecular dipole (Figure 4g). The synergistic effect of both phenomena working together can explain the decrease in the total dipole moment and thus polarity during the disassembly process.

The hypothesis that the pathway complexity ruling the system disassembly and reassembly strongly influences the material characteristics is supported by SAXS experiments conducted using a synchrotron light source. Following the work of Larik et al. [25], we studied the phototriggered reassembly using 520 nm light instead of the 450 nm light previously employed in microscopy experiments because the dye molecule was absent in these experiments. Data measured from a drop of disassembled gel (sol state) during a complete reassembly‐disassembly‐reassembly cycle irradiated over the whole sample volume (*bulk* irradiation) revealed distinct behaviors at each stage (Figure 5).

**FIGURE 5:**
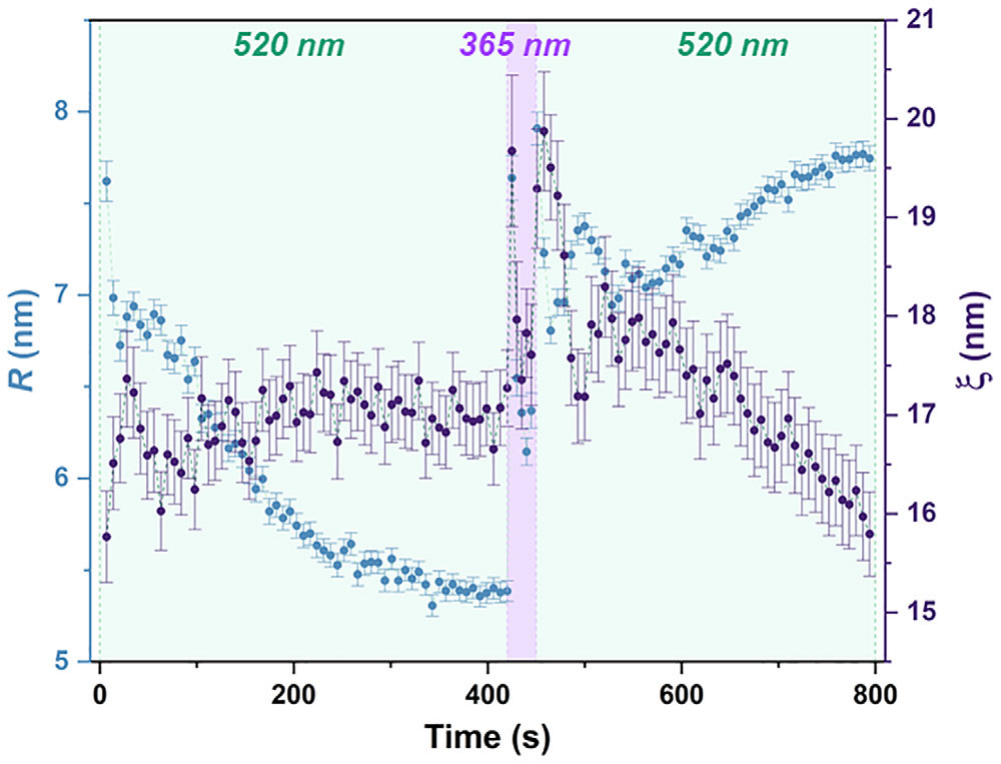
SAXS during **AAP** hydrogel photoreversion. Scatterer radius (*R*), and fractal correlation length (*ξ*) parameters obtained from modeled SAXS data of disassembled gel (sol state) during a complete reassembly‐disassembly‐reassembly cycle. Time intervals during bulk, 520 nm irradiation are back‐shaded in green, whereas the time interval with bulk, 365 nm‐irradiation is back‐shaded in purple.

During the first irradiation with 520 nm, the average radius of the scatterers decreases asymptotically while the fractal correlation length remained nearly constant. This behavior is consistent with a high nucleation rate driven by supersaturation, the primary driving force behind nucleation and growth. As a result, numerous small particles are formed without significant changes in long‐range structure, leaving the fractal correlation length unaltered. It is important to notice how this behavior is strikingly similar to what was observed during the GdL‐triggered supramolecular polymerization (Figure S7). Moreover, whereas both phenomena are not strictly start from the same system composition (GdL‐prepared sample initiates from a basic/anionic solution while the phototriggered sample comes from a neutral one), both lack the presence of any particle big enough to act like a nucleus from where the system may start to growth. Hence, in both cases a nucleation stage is needed to start the supramolecular polymerization.

Upon subsequent UV exposure to transform the gel into sol (time interval shaded in purple in Figure 5), both the average radius and the fractal correlation length increased sharply, seemingly contradicting the idea of full solubilization. This can be explained by partial dissolution of only the smallest nuclei, which shifts the size distribution toward larger particles. The resulting loss of interconnectivity between scatterers would also increase the fractal correlation length, as the density of the spanning network decreases. When 520 nm irradiation resumed, the system no longer followed its initial pathway. Instead of decreasing the average radius, it now increased while lowering the fractal correlation length. This shift indicates that growth now proceeds primarily by enlarging the remaining larger nuclei, those not fully dissolved during UV exposure, rather than by initiating new nucleation events [32, 33]. In this stage, coarsening effects dominate, where smaller particles dissolve, and their material feeds the growth of larger ones [34]. The initial supersaturation induced by the progressive acidification from GdL, which triggered the first nucleation burst, is no longer present, forcing the system to polymerize through a whole different pathway. The observation of small, interconnected crystalline particles giving rise to a fractal correlation length in SAXS experiments is in great agreement with the previously reported AFM images presented by Larik et al. [25]. In those images, it could be clearly identify the presence of different groups of small, well‐defined particles arranged in a dendritic way that arise from dried‐out solutions of hydrogel samples.

A notable observation from the obtained SAXS data is that all three irradiation cycles begin from the same minimal average radius of 7.6 nm. This persistence suggests that even under strong irradiation conditions, a fully soluble state of free, non‐interacting particles is never achieved. Instead, the system likely exists as a pre‐assembled network, where the gel structure arises from interparticle interactions between supramolecular small entities already present within the system. From this perspective, the gel is not a random aggregate but the result of a delicate balance between isomerization, solubilization, particle nucleation and growth, and colloidal interactions, all of which can be tuned by alternating UV and green light.

The idea that the system may be pre‐assembled in solution, even when fully composed of only *Z*‐**AAP** isomers, is strongly supported by MD simulations. For a system composed of this composition, simulations clearly show the spontaneous formation of supramolecular clusters with virtually no isolated molecules remaining in solution (Figure 4d). These clusters lack the highly ordered aromatic stacking typical of *E*‐**AAP** isomer assemblies (Figure 4a) because torsion in the azo bond's dihedral angle twists the molecule, preventing efficient π–π stacking. Nevertheless, the clusters are both spontaneous and stable, appearing within the first nanoseconds and persisting thereafter.

Based on these results, we can conclude that both photoinduced disassembly and reassembly pathways are mediated by a fragmentation mechanism where these fragments suffer strong changes in their polarity upon isomerization. Both FLIM and SAXS experiments suggest that these fragments reach a minimum size while increasing their dipolar moment until a solubility threshold is met (meaning the system's macroscopic properties are consistent with a homogeneous phase), but the supramolecular architectures are never completely lost (Figure 6). The presence of these soluble supramolecular architectures with strong polarity differences was not only experimentally detected for both isomers, but it is also supported by the spontaneous self‐assembly and the corresponding changes in the collective dipolar moment observed by MD simulations.

**FIGURE 6:**
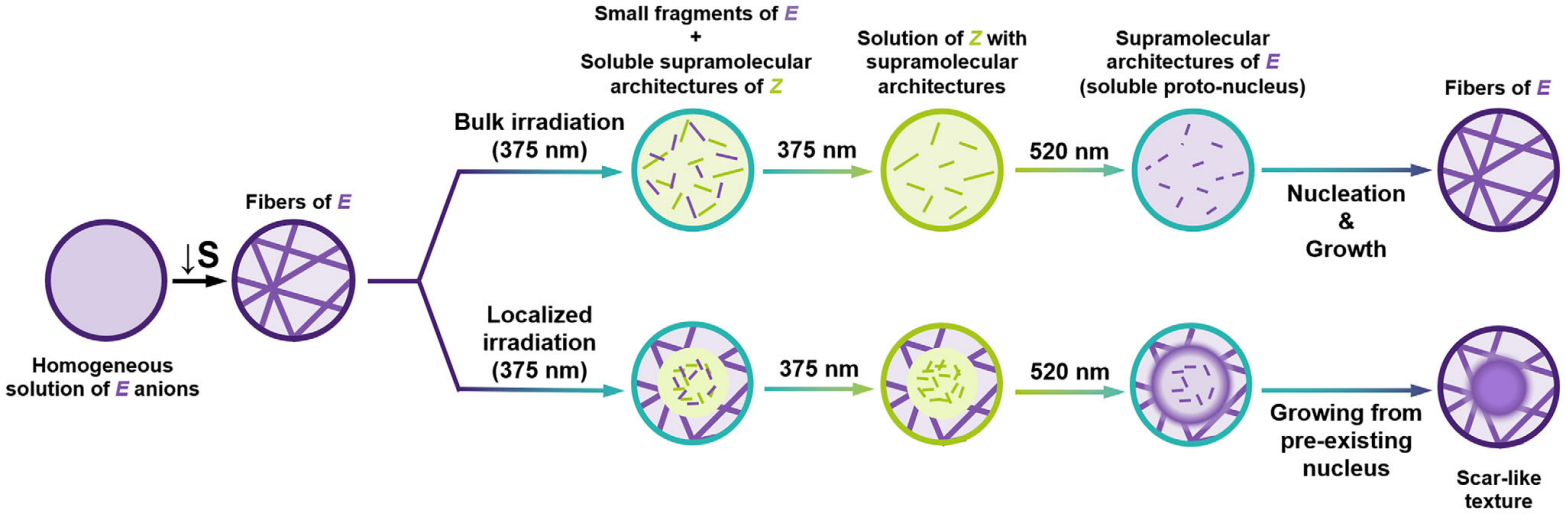
Proposed mechanism for phototriggered disassembly/reassembly of **AAP**. The scheme represents the mechanism involved in the supramolecular polymerization of a general self‐assembled photoreversible system triggered by a sharp decrease in the solubility (↓S) detailing the differences presented between whole‐sample irradiation (bulk, above) or localized volume irradiation (under).

Hence, we propose an overall mechanism for the photoreversible gelation of the model hydrogel in which supramolecular polymerization is governed by the interaction between pre‐assembled supramolecular clusters. Under the correct irradiation wavelength, these fragments change their morphology and polarity because of the substantial structural changes caused by isomerization. These changes, as a consequence, modify their complementarity and affinity, triggering either a fragmentation of a bulk, macroscopic fiber, or the cooperative reassembly of a solid phase by fragment deposition.

On the starting basic solution, from where the pH‐switched system is generated, all the molecules present in the system are freely diffusing and not interacting (or not in a substantial manner) due to the net negative charge of the *Z*‐**AAP** building blocks. Thereby, upon neutralizing the charge, polymerization is triggered, thus involving both nucleation and growth stages, as clearly depicted in SAXS experiments (Figure S7). In contrast, when the system starts polymerizing from a previously irradiated solution, the overall net charge of the system is not changed, but strong changes in polarity are also generated (Figure 4g). This change in the total dipolar moment will act as moving force of the precipitation in a similar way that neutralization does to the pH‐switched method, as both methodologies strongly diminish the solubility and allow the system to reach its threshold. But, in this scenario, the high number of undissolved fragments act as pre‐existing nuclei, as could be clearly seen during the photoinduced reassembly studied by SAXS (Figure 5). It is interesting to notice that, when the system starts from a neutrally charged *E*‐**APP** solution, it will present pre‐assembled structures, facilitating the formation of the compact and ordered nuclei required to develop the solid.

Moreover, during the phototriggered closing of the engraved hole (Figure 1c and Figure S3), one can observe the presence of numerous fragmented fibers on the side of the hole acting as multiple nucleation points from where fibers start to grow. From these points, polymerization proceeds and may start branching and fusing the generated fibrils, culminating in the formation of the dense, compact scar observed in the images. In both cases, the system is allowed to partially bypass the nucleation stage and develop a more continuous and intricate supramolecular network due to the presence of multiple starting points. Importantly, this pathway complexity explains the differences in the time required to obtain a gel‐like rheological response observed by Larik et al. [25] when comparing a freshly prepared sample to one irradiated during a whole disassembly‐reassembly cycle (c.a. 2 h for the fresh sample vs. c.a. 42′ for the phototreated one), supporting the critical role that nucleation points have in the features of the generated hydrogels.

Finally, it is interesting to point out how this model is consistent with previously reported observations of other authors regarding some unexpected characterization results. For instance, when Larik et al. perform irradiation‐dependent rheology experiments on this system, they notice that, in order to explain that the material seems to present a liquid response although the G′″/G′ ratio is above 1, they needed to propose the formation of a structured liquid. Remarkably, the existence of this structured‐liquid state is extremely consistent with our proposed mechanisms of fragmentation, where a fully free‐monomer state could not be achieved and the final solution is composed of multiple pre‐assembled supramolecular architectures that are capable of dynamically interact with each other.

Even more, this pathway complexity is observed by the authors ubiquitously over all the work. For instance, regarding gel morphology, the authors suggested that the variations observed for freshly prepared samples and the irradiated ones may arise from differences between “the aggregates in the sol *Z*‐2 PSS_Z_ state.” We propose that these suggested aggregates in the sol state are, in fact, the pre‐assembled fragments proposed by our mechanism. Thus, this model bridges the gap between the observed changes in the properties of samples prepared by different methods by explaining them in simple terms regarding of the presence or absence of pre‐assembled supramolecular structures.

## 3 Conclusions

Although multiple individual techniques have been proposed in recent years as a fully functional tool to study in detail the underlying process that rules supramolecular polymerization by self‐assembly, none of them have been able to depict a truly dynamical and time‐resolved description of the whole panorama with the required spatial resolution to distinguish the multiple supramolecular entities that may be involved. Moreover, when this required spatial resolution is achieved, it is usually detrimental to the time scale, in some cases even losing the whole dynamic dimension of the system due to the limitations of some sample preparations. Herein, by employing a multidisciplinary approach based on the great spatial and time resolution offered by confocal microscopy, the high‐contrast and molecular information of FLIM‐based imaging, and complementary structural information extracted from SAXS experiments and MD simulations, we were able to fully describe the whole phenomenon of disassembly and reassembly of a photoreversible hydrogel system in a comprehensive manner. Moreover, the great tridimensional spatial resolution of the confocal illumination technique allowed us to perform maskless photopatterning inside the gel matrix, opening up possibilities for developing novel microfluidic environments on biocompatible hydrogels that can be opened and closed at discretion by a non‐invasive long‐range stimulus.

Importantly, FLIM and SAXS results strongly complement each other to create a comprehensive picture of the self‐assembly mechanism across different scales that could not be obtained from either technique independently. On the one hand, FLIM microscopy allowed the direct observation of the system morphology, making it possible to compare the interconnectivity and topology resulting from different supramolecular polymerization processes. It also enabled us to track polarity changes in real time during disassembly and reassembly with high spatial resolution, providing crucial information on the changes in supramolecular organization induced by photoisomerization. On the other hand, SAXS experiments provided dynamic information on changes in the size distribution and fractal connectivity of the assemblies that could not be obtained by FLIM because of its spatial resolution limitations, whereas MD simulations offered a molecular‐level interpretation of their organization and polarity changes. The principal conceptual advance of this work therefore arises from the synergistic integration of these complementary observations, which allowed us to establish that phototriggered disassembly proceeds through fragmentation rather than complete solubilization and that the remaining fragments act as pre‐existing nuclei directing reassembly along a different pathway. Beyond elucidating the previously unresolved disassembly and reassembly mechanism of this particular hydrogel, the methodology presented here provides a broadly applicable strategy for investigating photoinduced transformations in supramolecular materials.

We presented herein a pathway complexity mechanism that supports recently published studies, indicating the importance of cooperative interactions between pre‐existing assemblies in solution as the starting point for macroscopic self‐assembled systems. In our case, the limit scenario of a fully dissolved system composed of neutral, non‐interacting particles seems never to be achieved, imitating a material that retains a ‘molecular memory’ via stable supramolecular clusters, preventing full solubilization. Moreover, here we show that the presence of these supramolecular entities in solution seems to be inevitable despite the extent of the illumination employed for the disassembly. The ubiquitous presence of these pre‐assembled supramolecular structures seems to be ruling the pathway complexity of this system by imposing a starting point from where supramolecular polymerization occurs.

## 4 Experimental Section

### 4.1 Synthetic Methods

The synthesis of **AAP** and its precursor was followed as described by Larik et al. [25]. **AQui** (9‐(azetidin‐1‐yl)‐5‐butyl‐4H‐benzo[*de*][2,6]naphthyridine‐4,6(5*H*)‐dione was prepared using our previously described method [26].

NMR spectra (^1^H and ^13^C) were performed in CDCl_3_ at room temperature on a Bruker‐400 spectrometer and high‐resolution mass spectrometry on a QTOF Compact (Bruker) spectrometer to ensure the identity of the product and its purity.

### 4.2 Gel Preparation Procedures

#### 4.2.1 Gel Preparation by pH Switch With Glucono‐δ‐Lactone (GdL)

1.03 mg (1 equiv.) of **AAP** was suspended in 50 µL of water and dissolved by the addition of 12 µL of aqueous sodium hydroxide solution (0.5 M, 1.5 equiv.). After full dissolution, 1 µL of an 1 mM **AQui** [26] solution in DMSO was added, and the solution was sonicated for *c.a*. 10 s to guarantee a homogeneous distribution of the dye before triggering the gelation. This solution was transferred to an uncoated Ibidi µ‐Slide 18 Well with glass bottom, and then, a fresh solution of GdL (2.14 mg (3 equiv.) in 38 µL of MiliQ water) was added to lower the pH. After about 1–2 h resting under ambient conditions and covered with aluminium foil to avoid any spurious light interaction, gels suitable for FLIM microscopy were obtained.

#### 4.2.2 Gel Preparation by pH Switch With Hydrochloric Acid

1.03 mg (1 equiv.) of **AAP** was suspended in 50 µL of water and dissolved by the addition of 12 µL of aqueous sodium hydroxide solution (0.5 M, 1.5 equiv.). After full dissolution, 1 µL of an 1 mM **AQui** [26] solution in DMSO was added, and the solution was sonicated for *c.a*. 10 s to guarantee a homogeneous distribution of the dye before triggering the gelation. This solution was transferred to an uncoated Ibidi µ‐Slide 18 Well with glass bottom, and then, 12 µL of HCl 1 M (3 equiv.) were added to lower the pH. Although the addition of the HCl solution seems to instantly form a hydrogel, the system was also let to rest to about 1–2 h under ambient conditions and covered with aluminum foil to allow gelation to occur and to guarantee identical sample preparation conditions for both types of gel.

### 4.3 Fluorescence Lifetime Microscopy (FLIM) and Image Analysis

FLIM imaging was performed using a confocal microscope (Abberior Instruments GmbH, Heidelberg, Germany) equipped with a Multiharp 150 4N time‐correlated single‐photon counting (TCSPC) module (PicoQuant GmbH, Germany). The system was configured with a UPlanSApo 100× oil‐immersion objective (NA 1.4), and the pinhole was set to 1 Airy unit. The samples were excited with a 561 nm pulsed laser for data acquisition, working at 15 MHz for a TCSPC time window of 66 ns with a time resolution of 80 ps/channel.

Disassembly and reassembly processes were triggered employing two irradiation lasers: the 375 nm laser at 10% power for a period of ∼0.3 to 1 ms per pixel for the *E*‐to‐*Z* transition (disassembly process) and 450 nm laser at 90% power for a period of ∼5 to 7 ms per pixel for the *Z*‐to‐*E* reassembly process. If needed, multiple irradiation cycles with these exact same parameters were performed in order to account for the full disassembly or reassembly of the system. The emission from **AQui** was collected on an avalanche photodiode (APD) after a 580–630 nm bandpass filter. FLIM data acquisition was done with a pixel size of 50 nm and a 200 µs dwell time per pixel. FLIM image analysis was done with SymPhoTime 64 (PicoQuant) software by fitting the fluorescence decay traces obtained in each individual pixels to a single exponential decay function, using the maximum likelihood estimator (MLE) for parameter optimization, which yields the correct parameter set for low count rates. To quantify the changes in polarity produced during the disassembly and reassembly process, histograms of the fitted decay time were extracted from selected regions of interest containing the irradiated volume.

### 4.4 Computational Methodology

MD simulations were carried out using GROMACS 2023 software [35]. This force field has been extensively utilized in diverse supramolecular simulations [36] due to its versatility. The following workflow was employed in the overall dynamic simulation analysis: the three‐dimensional structure of **AAP** in both, *E* and *Z* configuration, was generated using Avogadro software [37] and their respective topology file was computed using the online server SwissParams [38] with the CHARMM27 force field [39]. Geometry optimization of both, *E* and *Z* isomers were done employing ORCA6 software [40] with B3LYP functional [41, 42, 43], def2‐TZVP basis set, D4 dispersion correction and taking advantage of the RIJCOSX approximation to speed up calculation. These geometry optimizations were done to check for the best dihedral angle for the C–N = N–C bond in both configurations. These angular values were constrained in the MD simulation to avoid undesired isomerization during system stabilization.

All the studied system was composed of a total amount of 42 molecules in a 12·12·12 nm cubic box surrounded with water molecules (SPC/E model) [43], giving a final concentration equal to the employed experimental concentration (4·10^−2^ M). When mixtures of different *E* or *Z* proportions were studied, the total amount of 42 molecules was kept constant. Due to the low concentration of dye in microscopy studies, only 1 molecule of **AQui** was introduced to the system when studying the dye‐molecule interaction, although this implied a higher concentration than that used in the microscopy experiments.

After preparing these simulation boxes, energy minimization was performed using the steepest descent algorithm. To ensure a strictly minimized system the cut‐off value for the maximum tolerable force was kept at less than 800 kJ/mol, and the number of steps was increased up to 100.000. These energy‐minimized structures were later equilibrated at both constant volume and pressure. The LINCS algorithm was utilized to constrain only the bond lengths involving hydrogen. To achieve the desired temperature of 298 K, the modified Berendsen thermostat known as V‐rescale was employed for 300 ps. To achieve a pressure of 1 bar, the Berendsen barostat algorithm was utilized for 300 ps. Compressibility was adjusted to 4.5·10^−5^. Particle Mesh Ewald model was employed to account for long‐range interactions, with 1 nm cut‐off for both, short‐range electrostatic and van der Waals interactions. After the equilibration phases, the simulations were carried out for at least 100 ns.

For the analysis of the disassembly process starting from the crystal structure, a portion of 108 molecules was constructed from the Crystallographic Information File (CCDC Deposition Number: 1977104), boxed on a cubic volume of 12·12·12 nm, solvated with water, and their positions were restrained for all the minimization and stabilization processes until the beginning of the simulation.

To quantify the total dipolar moment of the supramolecular arrangement developed during the molecular dynamics simulation the “dipole” module, included in the GROMACS package, was employed. To avoid any spurious contribution of solvent or ions present in the simulation, an index containing only **AAP** molecules was used. When studying the changes in the total molecular dipole of isomer's mixtures, box plots were calculated considering the dipole moment computed for the last 20 ns of each simulation, and the plots were done with the Origin9 software.

### 4.5 SAXS Measurement

In situ Small Angle x‐ray Scattering (SAXS) experiments were performed at the BL11 NCD‐SWEET beamline at the ALBA Synchrotron Light Source (Barcelona, Spain), under project ID 20250340041.

Samples were measured on quartz capillary tubes, and all the measurements were carried out at room temperature. The x‐ray beam was operated at 15 keV, and the distance between the sample and detector was set to 6700 mm. A Pilatus 1 M detector (Dectris, Baden, Switzerland) was employed to capture the two‐dimensional patterns, capturing consecutive images with a 5‐second exposure time. The number of images acquired was selected according to the experiment performed (kinetics studies or characterization during light irradiation). The one‐dimensional patterns were obtained through azimuthal integration using the pyFAI library [44], and the intensity was expressed as a function of *q* (scattering momentum transfer: 𝑞 = 4*(𝜋/𝜆)*𝑠𝑖𝑛(𝜃)). For the irradiation experiments two Thorlabs high‐power LED, a UV (365 nm, M365L2) and a green (520 nm, M530L3), connected to independent DC40 drivers, were employed. Both LED light sources were capped with an adjustable collimation adapter (SM1U25‐A, AR Coating: 350–700 nm) that aimed to focus the beam on the sample holder. The light source and the drivers were allocated inside the experimental hutch, and both LEDs were positioned at the same distance of the sample holder. Each of the light sources was employed at full power, and the distance to the sample was optimized for each experiment, guaranteeing a minimum time for full transformation followed by visual inspection of about 30s. This allowed us to perform at least 6 independent data acquisitions of 5 s of x‐ray irradiation each. Sensitivity of the sample towards different wavelengths (due to differences in the molar absorptivity) was also taken into consideration when positioning each LED. All sample manipulation was done over indirect red‐light illumination to avoid any undesired isomerization of the sample before starting the experiments.

The scattering intensity was interpreted in terms of a mass fractal system built from small spherical subunits assembling into larger supramolecular structures with a non‐compact architecture. The total intensity was modeled under the local monodispersity approximation:

$$I\left(q\right)=K\cdot S\left(q\right)\cdot P\left(q\right)+\mathrm{back}$$

where K and back are scalar, and S(q) is the mass fractal structure factor, in which *D* is the fractal dimension and *ξ* is the fractal cutoff length, also referred to as the fractal correlation length [45].

$$\begin{array}{cc} S\left(q\right) & =1+\frac{D\;\cdot \;\Gamma \left(D-1\right)\;\cdot \;sin\left(\left(D-1\right)\;arctan\left(q\xi \right)\right)}{{\left(1+\frac{1}{q^{2}\xi^{2}}\right)}^{\left(D-1\right)/2}\;}\;\cdot \\ & \;{\left(q\sqrt{5/3}R_{g}\right)}^{-D} \end{array}$$

The radius of gyration *Rg* of the individual subunit was obtained using the semi‐empirical Guinier–Porod model: [46]

$$P\left(q\right)=\left\{\begin{array}{c} \;exp\left(-\frac{{\left(q\;R_{g}\right)}^{2}}{3\;}\right)\;if\;q\;\leq \;\frac{\sqrt{6}}{R_{g}}\\ \;exp\left(-2\right)\;\cdot \;36\;\cdot \;{\left(R_{g}q\right)}^{-4}\;if\;q\;>\;\frac{\sqrt{6}}{R_{g}} \end{array}\right.$$

In this model, the form factor was simplified assuming a compact spherical geometry for the scattering subunit.

### 4.6 Powder X‐Ray Diffraction Experiments

Hydrogels (40 mM) from AAP with two different acidification methods (GdL and HCl) were prepared as described above and let to rest for 12 h. After that, the solid phase was separated by centrifugation, washed two times with distilled water, and let to dry at room temperature overnight. Samples were milled before measurements to guarantee a more homogeneous size and shape distribution, and to avoid any artifacts that may arise due to the special orientation of the crystals.

Powder x‐ray diffraction data were obtained at room temperature on a Bruker D2 PHASER diffractometer using Cu Kα radiation (λ = 1.5418 Å), collecting from 4 to 40° in the 2θ range, with steps of 0.02° and time‐per‐step of 0.5 s. Milled samples were measured using a zero‐background silicon sample holder. Background subtraction was performed using WinPLOTR software [47] included in the FullProf suite [48].

### 4.7 Kinetic NMR Experiments

The kinetics of the self‐assembly process in the presence and absence of **AQui** was monitored by solution‐state ^1^H NMR measurements using a Bruker‐500 MHz spectrometer. To monitor the starting point of the gelation process, the gel mixture was prepared in a vial and transferred to an NMR tube for its immediate measurement. The 1 mL of gel was prepared by suspending 10.3 mg of **AAP** in 500 µL of D_2_O and subsequently dissolved by addition of 2 equivalents of NaOH from a 0.5 M solution in D_2_O. 10 µL of 1 mM solution in DMSO were added for the experiment with **AQui**. No **AQui** solution was added to the control experiment. The final volume was completed with D_2_O containing 3 equivalents of GdL. The kinetic ^1^H NMR measurements were performed on the prepared solutions immediately after shimming (t = 0). One spectrum was taken every 5 min for 80 min at room temperature. For date representation, all spectra have been referenced to the solvent peak.

## Conflicts of Interest

The authors declare no conflicts of interest.

## Supporting information




**Supporting File 1**: smll75208‐sup‐0001‐SuppMat.docx.

**Supporting File 2**: smll75208‐sup‐0002‐VideoS1.mp4.

**Supporting File 3**: smll75208‐sup‐0003‐VideoS2.mp4.

**Supporting File 4**: smll75208‐sup‐0004‐VideoS3.mp4.

**Supporting File 5**: smll75208‐sup‐0005‐VideoS4.mp4.

## Data Availability

The datasets generated during this study are publicly available ohttps://doi.org/10.5281/zenodo.21469626.
