# Supplementary material for: Unraveling Pathway Complexity in Photoreversible Supramolecular Hydrogels: Real‐Time Visualization and Sub‐Micrometric Photopatterning
Source: Small. 2026 Aug 16;22(58):e75208. doi: 10.1002/smll.75208 (PMC13676842; doi:10.1002/smll.75208)
Supplement: Supplementary file 1 [file SMLL-22-e75208-s003.docx]

Supporting Information

Unraveling Pathway Complexity in Photoreversible Supramolecular Hydrogels: Real-Time Visualization and Sub-Micrometric Photopatterning

Federico Movilla, ^1^ Consuelo Ripoll, ^1^ Cristián Huck-Iriart,^2^ Juan A. González-Vera,^1^ Angel Orte,^1^ and Rafael Contreras-Montoya*^1^

F. Movilla, C. Ripoll, A. Orte, J. A. González-Vera and R. Contreras-Montoya

Nanoscopy-UGR Laboratory. Departamento de Fisicoquímica, Unidad de Excelencia de Química Aplicada a Biomedicina y Medioambiente, Facultad de Farmacia

Universidad de Granada

Campus Cartuja, 18071, Granada, Spain
E-mail: rcm@ugr.es

C. Huck-Iriart
ALBA Synchrotron Light Source, Carrer de la Llum 2–26, Cerdanyola del Vallès, 08290 Barcelona, Spain

Index

[Crystallographic characterization of samples obtained by different acidification methods	3](#_Toc235096482)

[Evaluation of the influence of AQui on the self-assembly process.	3](#_Toc235096483)

[Photoreversibility observed by confocal microscopy	4](#_Toc235096484)

[Disassembly and reassembly of a hydrogel sample obtained with HCl	4](#_Toc235096485)

[Photopatterning characterization	4](#_Toc235096486)

[Control experiments for FLIM microscopy	6](#_Toc235096487)

[SAXS measurement	7](#_Toc235096488)

[SAXS measurement on the original basic solution previous the addition of GdL.	7](#_Toc235096489)

[DFT calculation	7](#_Toc235096490)

[Cartesian coordinates of optimized structures	7](#_Toc235096491)

[Molecular dynamics simulations	8](#_Toc235096492)

[Molecular dynamics parameter file	8](#_Toc235096493)

[Energy minimization parameters	8](#_Toc235096494)

[Ions insertion parameters	9](#_Toc235096495)

[NVT stabilization parameters	9](#_Toc235096496)

[NPT stabilization parameters	10](#_Toc235096497)

[Molecular dynamics simulation parameters	11](#_Toc235096498)

# Crystallographic characterization of samples obtained by different acidification methods

**Figure S1.** Powder X-ray diffraction patterns measured for the solid phase of AAP gel samples obtained by HCl acidification (yellow) and glucono-δ-lactone (orange). The calculated pattern diffraction simulated from the single crystal structures of the neutral form of AAP (violet) and NaCl (gray) are included for comparation. Unmatched signals that may arise from NaCl contamination in the **AAP-HCl** sample are highlighted with a star.

# Evaluation of the influence of AQui on the self-assembly process.

**Figure S2.** Kinetics of gel formation (40mM in D_2_O) in the absence (left) and presence (right) of AQui (10 µM) followed by NMR spectroscopy.

# Photoreversibility observed by confocal microscopy

## Disassembly and reassembly of a hydrogel sample obtained with HCl

**Figure S3.** Confocal images of **AQui** (50µM, λ_ex_ = 561nm) in a HCl-prepared AAP hydrogel, during **(a)** phototriggered disassembly and **(b)** reassembly. Every frame was taken after 3’of irradiation with the corresponding 375 nm or 450 nm light source.

# Photopatterning characterization

**Figure S4**. **a,** Area selected to perform the qualitative texture analysis. In green and orange are highlighted the same axis that are represented in the sequential cross-section cascade. **b,** Sequential cross-section cascade plot representing the changes in intensity observed in the area highlighted in (a).

**Figure S5**. **a,** Detail of the cross-sections selected for width determination of the photocarved area. **b,** Intensity data (filled circles) and adjacent-averaged (15 points) smoothed line representation used for width determination. **c,** Histogram of the widths measured.

# Control experiments for FLIM microscopy

**Figure S6.** FLIM images performed over a freshly gelificated sample (above) and a disassembled portion (below) before and after irradiating uninterruptedly with 561 nm for 10 minutes. The pseudocolor scale indicates the average τ_f_ of AQui and its relationship with the environment polarity is indicated for clarity.

# SAXS measurement

## SAXS measurement on the original basic solution previous the addition of GdL.

**Figure S7**. SAXS during pH-induced supramolecular polymerization. Scatterers radius (R), fractal correlation length (ξ) and fractal dimension (D_Fr_) parameters obtained from modeled SAXS data for a GdL-prepared AAP hydrogel during supramolecular polymerization.

# DFT calculation

## Cartesian coordinates of optimized structures

| ***Z* isomer** | | | |  | ***E* isomer** | | | |
| --- | --- | --- | --- | --- | --- | --- | --- | --- |
| **O** | 5.658789 | 14.156701 | 8.273927 |  | **O** | -0.577459 | 10.431735 | 11.350297 |
| **O** | 3.458815 | 14.137468 | 7.847981 |  | **O** | -0.060319 | 11.873178 | 9.714817 |
| **N** | 2.516382 | 8.749393 | 5.032177 |  | **N** | 2.72329 | 8.070372 | 5.609169 |
| **N** | 4.458537 | 7.908779 | 2.231766 |  | **N** | 4.297953 | 7.923223 | 2.494233 |
| **N** | 4.763879 | 9.233082 | 2.237078 |  | **N** | 3.976868 | 9.251459 | 2.452557 |
| **N** | 2.60907 | 9.463415 | 6.054368 |  | **N** | 2.188565 | 9.035236 | 6.216134 |
| **C** | 3.788502 | 10.184998 | 6.399417 |  | **C** | 1.640531 | 8.710974 | 7.479725 |
| **C** | 3.632696 | 7.569997 | 3.233054 |  | **C** | 3.885824 | 7.329909 | 3.620711 |
| **C** | 4.742338 | 12.183607 | 7.351919 |  | **C** | 0.51889 | 9.570366 | 9.442381 |
| **C** | 3.635129 | 11.474241 | 6.892394 |  | **C** | 1.089102 | 9.774836 | 8.18727 |
| **C** | 4.158377 | 9.755346 | 3.293319 |  | **C** | 3.351942 | 9.520755 | 3.583717 |
| **C** | 3.433485 | 8.734332 | 3.979741 |  | **C** | 3.261712 | 8.332428 | 4.376162 |
| **C** | 4.531292 | 13.570567 | 7.837196 |  | **C** | -0.053669 | 10.741372 | 10.151914 |
| **C** | 6.002218 | 11.581762 | 7.358653 |  | **C** | 0.497448 | 8.288762 | 9.994646 |
| **C** | 5.038116 | 7.035059 | 1.229434 |  | **C** | 4.948614 | 7.317647 | 1.349601 |
| **H** | 5.506298 | 7.661442 | 0.475531 |  | **H** | 5.657381 | 8.031188 | 0.935366 |
| **H** | 4.262154 | 6.427008 | 0.765863 |  | **H** | 4.213705 | 7.052184 | 0.587393 |
| **H** | 5.787921 | 6.381012 | 1.677577 |  | **H** | 5.478726 | 6.422712 | 1.665488 |
| **C** | 4.211472 | 11.226564 | 3.527042 |  | **C** | 2.866292 | 10.896749 | 3.875372 |
| **H** | 4.926245 | 11.487129 | 4.309347 |  | **H** | 3.324953 | 11.28869 | 4.785065 |
| **H** | 3.237722 | 11.610036 | 3.83388 |  | **H** | 1.787368 | 10.903173 | 4.040334 |
| **H** | 4.511068 | 11.729362 | 2.607787 |  | **H** | 3.105017 | 11.556844 | 3.041246 |
| **C** | 5.045505 | 9.573915 | 6.42582 |  | **C** | 1.615157 | 7.425613 | 8.037746 |
| **C** | 3.123057 | 6.195029 | 3.460814 |  | **C** | 4.084127 | 5.891178 | 3.927876 |
| **H** | 2.752473 | 5.752922 | 2.534142 |  | **H** | 3.58669 | 5.258654 | 3.188943 |
| **H** | 2.313598 | 6.222076 | 4.188425 |  | **H** | 3.672365 | 5.669261 | 4.909796 |
| **H** | 3.908037 | 5.540672 | 3.849915 |  | **H** | 5.14517 | 5.630695 | 3.926929 |
| **C** | 6.141125 | 10.274282 | 6.908418 |  | **C** | 1.046671 | 7.224811 | 9.285199 |
| **H** | 7.112329 | 9.797008 | 6.932839 |  | **H** | 1.027727 | 6.230297 | 9.712734 |
| **H** | 5.159993 | 8.555471 | 6.08125 |  | **H** | 2.038879 | 6.597746 | 7.487516 |
| **H** | 2.655326 | 11.931576 | 6.90599 |  | **H** | 1.107062 | 10.76555 | 7.754234 |
| **H** | 6.861877 | 12.124453 | 7.723637 |  | **H** | 0.055583 | 8.122652 | 10.966393 |
| **H** | 5.440476 | 15.055487 | 8.573199 |  | **H** | -0.931014 | 11.245078 | 11.748083 |

# Molecular dynamics simulations

## Molecular dynamics parameter file

### Energy minimization parameters

| integrator | = | steep |
| --- | --- | --- |
| emtol | = | 800 |
| emstep | = | 0.01 |
| nsteps | = | 100000 |
|  |  |  |
| nstlist | = | 1 |
| cutoff-scheme | = | Verlet |
| ns_type | = | grid |
| coulombtype | = | P3M-AD |
| rcoulomb | = | 1 |
| rvdw | = | 1 |
| pbc | = | xyz |

### Ions insertion parameters

| integrator | = | steep |
| --- | --- | --- |
| emtol | = | 1000 |
| emstep | = | 0.01 |
| nsteps | = | 50000 |
|  |  |  |
| nstlist | = | 1 |
| cutoff-scheme | = | Verlet |
| ns_type | = | grid |
| coulombtype | = | cutoff |
| rcoulomb | = | 1 |
| rvdw | = | 1 |
| pbc | = | xyz |

### NVT stabilization parameters

| integrator | = | md |
| --- | --- | --- |
| nsteps | = | 300000 |
| dt | = | 0.001 |
|  |  |  |
| nstxout | = | 500 |
| nstvout | = | 500 |
| nstenergy | = | 500 |
| nstlog | = | 500 |
|  |  |  |
| continuation | = | no |
| constraint_algorithm | = | lincs |
| constraints | = | h-bonds |
| lincs_iter | = | 1 |
| lincs_order | = | 4 |
|  |  |  |
| cutoff-scheme | = | Verlet |
| ns_type | = | grid |
| nstlist | = | 10 |
| rcoulomb | = | 1 |
| rvdw | = | 1 |
| DispCorr | = | EnerPres |
|  |  |  |
| coulombtype | = | PME |
| pme_order | = | 4 |
| fourierspacing | = | 0.16 |
|  |  |  |
| tcoupl | = | V-rescale |
| tc-grps | = | System |
| tau_t | = | 0.1 |
| ref_t | = | 298 |
|  |  |  |
| pcoupl | = | no |
|  |  |  |
| pbc | = | xyz |
|  |  |  |
| gen_vel | = | yes |
| gen_temp | = | 298 |
| gen_seed | = | -1 |

### NPT stabilization parameters

| integrator | = | md |
| --- | --- | --- |
| nsteps | = | 300000 |
| dt | = | 0.001 |
|  |  |  |
| nstxout | = | 500 |
| nstvout | = | 500 |
| nstenergy | = | 500 |
| nstlog | = | 500 |
|  |  |  |
| continuation | = | yes |
| constraint_algorithm | = | lincs |
| constraints | = | h-bonds |
| lincs_iter | = | 1 |
| lincs_order | = | 4 |
|  |  |  |
| cutoff-scheme | = | Verlet |
| ns_type | = | grid |
| nstlist | = | 10 |
| rcoulomb | = | 1 |
| rvdw | = | 1 |
| DispCorr | = | EnerPres |
|  |  |  |
| coulombtype | = | PME |
| pme_order | = | 4 |
| fourierspacing | = | 0.16 |
|  |  |  |
| tcoupl | = | V-rescale |
| tc-grps | = | System |
| tau_t | = | 0.1 |
| ref_t | = | 298 |
|  |  |  |
| pcoupl | = | Parrinello-Rahman |
| pcoupltype | = | isotropic |
| tau_p | = | 2 |
| ref_p | = | 1 |
| compressibility | = | 4.50E-05 |
| refcoord_scaling | = | com |
|  |  |  |
| pbc | = | xyz |
|  |  |  |
| gen_vel | = | no |

### Molecular dynamics simulation parameters

| integrator | = | md |
| --- | --- | --- |
| nsteps | = | 1E+08 |
| dt | = | 0.002 |
|  |  |  |
| nstxout | = | 0 |
| nstvout | = | 0 |
| nstfout | = | 0 |
| nstenergy | = | 50000 |
| nstlog | = | 50000 |
| nstxout-compressed | = | 50000 |
| compressed-x-grps | = | System |
|  |  |  |
| continuation | = | yes |
| constraint_algorithm | = | lincs |
| constraints | = | h-bonds |
| lincs_iter | = | 1 |
| lincs_order | = | 4 |
|  |  |  |
| cutoff-scheme | = | Verlet |
| ns_type | = | grid |
| nstlist | = | 10 |
| rcoulomb | = | 1 |
| rvdw | = | 1 |
|  |  |  |
| coulombtype | = | PME |
| pme_order | = | 4 |
| fourierspacing | = | 0.16 |
|  |  |  |
| tcoupl | = | V-rescale |
| tc-grps | = | System |
| tau_t | = | 0.1 |
| ref_t | = | 298 |
|  |  |  |
| pcoupl | = | Parrinello-Rahman |
| pcoupltype | = | isotropic |
| tau_p | = | 2.00E+00 |
| ref_p | = | 1 |
| compressibility | = | 4.50E-05 |
|  |  |  |
| pbc | = | xyz |
|  |  |  |
| DispCorr | = | EnerPres |
|  |  |  |
| gen_vel | = | no |
